# Pharmacokinetics and Metabolism Study of Deep-Sea-Derived Butyrolactone I in Rats by UHPLC–MS/MS and UHPLC–Q-TOF-MS

**DOI:** 10.3390/md20010011

**Published:** 2021-12-22

**Authors:** Liang Wu, Chun-Lan Xie, Xian-Wen Yang, Gang Chen

**Affiliations:** 1 Department of Pharmaceutical Analysis, School of Pharmacy, Fudan University, Shanghai 201203, China; yhdhospital@163.com; 2 Key Laboratory of Marine Biogenetic Resources, Third Institute of Oceanography, Ministry of Natural Resources, 184 Daxue Road, Xiamen 361005, China; xiechunlanxx@163.com

**Keywords:** butyrolactone I, food allergy, metabolism, pharmacokinetics

## Abstract

Butyrolactone I (BTL-I) is a butanolide isolated from the deep-sea-derived fungus, *Aspergillus* sp. It provides a potential new target for the prevention and treatment of food allergies. This study aimed to investigate the metabolic and pharmacokinetic profile of BTL-I in rats. The metabolic profiles were obtained by UHPLC–Q-TOF-MS. As a result, eleven metabolites were structurally identified, and the proposed metabolic pathways of BTL-I were characterized. The main metabolites were the oxidative and glucuronidative metabolites. In addition, a sensitive UHPLC–MS/MS method was established for the quantitation of BTL-I in rat plasma (LOQ = 2 ng/mL). The method was fully validated and successfully applied to the pharmacokinetic study of BTL-I in rats after oral administration or intravenous administration. The oral bioavailability was calculated as 6.29%, and the maximum plasma concentrations were 9.85 ± 1.54 ng/mL and 17.97 ± 1.36 ng/mL for intravenous and intragastric dosing groups, respectively.

## 1. Introduction

Food allergies are an immune-mediated adverse reaction to food [1,2]. Food allergies to cow’s milk and eggs have become prevalent among young children over the past 20 years [3,4]. Currently, the only effective way to prevent allergic reactions to food is to strictly avoid the allergen of concern, and the recognized treatment for severe allergic reactions is epinephrine [5]. Consequently, providing improved therapeutic options has become an important avenue in food allergy research. The exploitation of small-molecule inhibitors is one important research direction for anti-food allergy studies, due to their diverse chemical structures [6]. There is certain evidence from human and animal studies that basophils and mast cells are key effector cells that contribute to allergic reactions to foods [7]. It has been reported that several compounds from marine microorganisms could partially inhibit degranulation in IgE-mediated mast cell responses [8,9].

Butyrolactone I (BTL-I) is a butanolide first reported in 1997 [10]. It showed an anti-neuroinflammatory effect on lipopolysaccharide (LPS)-induced microglia cells by inhibiting the phosphorylation of p65 and IκB [11]. Moreover, BTL-I could inhibit cyclin-dependent kinases (CDK), including CDK1 and CDK5 [12,13]. In a previous study, we obtained this compound from a deep-sea-derived *Aspergillus* sp. We found that it exhibited significant anti-food allergic activities in vitro and in vivo [14]. Therefore, BTL-I has additive or synergistic therapeutic potential in diseases with multi-factorial etiologies, and provides a potential new insight to the prevention and treatment of food allergy diseases [15].

Although the activities were studied extensively, the pharmacokinetics and metabolism of BTL-I remain unknown. Pharmacokinetics and metabolism are significantly important in the drug discovery and development processes, not only for supporting toxicity but also for optimizing the drug candidate [16]. Pharmacokinetics are useful for obtaining information to help understand the molecular basis of pharmacological activity, for treatment scheme selection, and for more effective drug application [17]. Therefore, it was meaningful to obtain the pharmacokinetic and metabolism profiles of the drug, to better comprehend how effective it could be. Previous studies have investigated the metabolism of BTL-I in rat urine and feces [18], in which ten phase I metabolites in rat feces were isolated and identified. However, the metabolites distributed in rat blood and bile were unknown.

In the present study, a rapid and sensitive LC–MS/MS method was developed and validated for the determination of BTL-I in rat plasma. A reliable method was subsequently applied to investigate the pharmacokinetic behaviors of BTL-I in rats after intravenous and oral administration. Moreover, the metabolism of BTL-I in vivo was investigated using an ultra-high-performance liquid chromatography–quadrupole time-of-flight mass spectrometry (UHPLC–Q-TOF-MS) method.

## 2. Results and Discussion

### 2.1. Metabolism Study of BTL in Rats

#### 2.1.1. Fragmentation of BTL-I Standard

**Figure 1:**
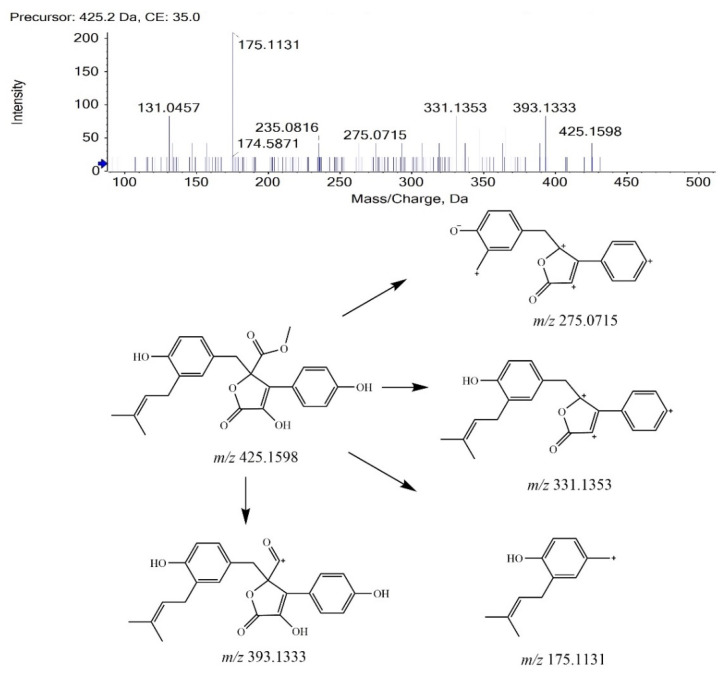
MS^2^ spectrum of BTL-I and its fragmentation pathways.

BTL-I was eluted at 13.88 min under the current chromatographic condition. The MS^2^ fragmentation pattern of BTL-I was studied for a better understanding of the MS^2^ fragmentation patterns of the metabolites. In positive ion mode, BTL-I showed a protonated molecular ion [M + H]^+^ at *m/z* 425.1598 (C_24_H_25_O_7_^+^). The MS^2^ fragmentation of the parent ion showed three predominant fragment ions at *m/z* 393.1333 (C_23_H_21_O_6_^+^), *m/z* 331.1353 (C_23_H_19_O_33_^+^), *m/z* 175.1131(C_12_H_15_O^+^). One minor ion at *m/z* 275.0715 (C_18_H_11_O_33_^+^) was also observed. The proposed fragmentation pathways are shown in Figure 1. These fragment ions can be used as characteristic ions for the identification of metabolites.

#### 2.1.2. Analysis of BTL-I Metabolites

By comparison with the blank samples, precursor ions uniquely found in BTL-I dosed samples were considered as potential metabolites, and then used for subsequent MS^2^ analysis. Eleven potential metabolites were observed and structurally identified based on their MS and MS^2^ spectra.

**Figure 2:**
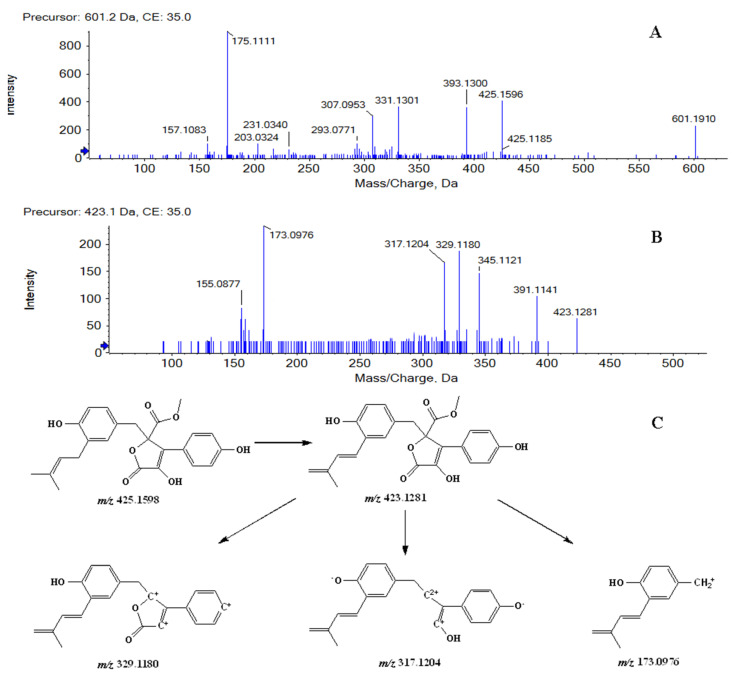
MS^2^ spectrum of M1, M2 (**A**), and M3, M4, M5, M6 (**B**), along with the fragmentation pathway of M3, M4, M5, M6 (**C**).

Metabolites M1 and M2 were detected at 11.08, 11.87 min, respectively. In positive ion mode, both of them showed the protonated molecular ion [M + H]^+^ at *m/z* 601.1910 (−0.7 ppm, element composition C_30_H_33_O_13_^+^), 176 *m/z* units higher than that of BTL-I, suggesting that they were glucuronide conjugates of BTL-I. The MS^2^ fragmentation (Figure 2A) showed fragment ions at *m/z* 425.1596, 393.1300, 331.1301, 175.1111. Therefore, Metabolites M1 and M2 were identified as the glucuronide conjugates of BTL-I.

Metabolites M3, M4, M5 and M6 were eluted at 8.42, 9.19, 10.82, 11.63 min, respectively. All of them showed the protonated molecule [M + H]^+^ at *m/z* 423.1440 (−1.0 ppm, element composition C_24_H_23_O_7_^+^), 2 *m/z* units lower than that of BTL-I. In the product ion scan (Figure 2B), product ions at *m/z* 391.1141, 345.1099, 329.1180, 317.1204 and 173.0976 were observed. The proposed fragmentation pathway was shown in Figure 2C. Therefore, metabolites M3, M4, M5 and M6 were tentatively identified as the dehydrogenate metabolites of BTL-I.

Metabolite M7 was eluted at the retention time of 11.17 min. It showed a protonated molecule [M + H]^+^ at *m/z* 443.1698 (−1.8 ppm, element composition C_24_H_27_O_8_^+^), 18 *m/z* units higher than that of BTL-I. Therefore, M7 was thought to be conjugated with water. 

The retention times of metabolites M8 and M9 were 9.38, and 11.71 min, respectively. Both of them showed the same protonated molecule [M + H]^+^ at *m/z* 411.1443 (−0.2 ppm, element composition C_23_H_23_O_7_^+^). In the product ion scan of M8 (Figure S3A), product ions at *m/z* 275.0743 and 175.1133 were observed. In the product ion scan of M9 (Figure S3B), product ions at *m/z* 393.1261, 331.1387, 275.0768 and 175.1133 were observed. Therefore, metabolites M8 and M9 were identified as the demethylation metabolites of BTL-I. The demethylation site of M9 was thought to be the methyl of ester linkage. 

Metabolite M10 was eluted at 13.03 min. It showed the protonated molecule [M + H]^+^ at *m/z* 441.1525 (−5.4 ppm, element composition C_24_H_25_O_8_^+^), 16 *m/z* units higher than that of BTL-I. At the further product ion scan (Figure S4), product ion at *m/z* 363.1112 was observed. Therefore, metabolite M10 was identified as an oxidate metabolite of BTL-I. According to the previous study [18], M10 was supposed as 7’’S-hydroxy-9’’-ene-butyrolactone I or 7’’R-hydroxy-9’’-ene-butyrolactone I.

Metabolite M11 was eluted at 11.96 min. It showed the protonated molecule [M+H]^+^ at *m/z* 457.1506 (1.8 ppm, element composition C_24_H_25_O_9_^+^), 16 *m/z* units higher than that of M10. Therefore, metabolite M11 was identified as a dioxidation metabolite of BTL-I. According to the previous study [18], M11 was supposed as 7’’S,8’’R-dihydroxy-aspernolide E or 7’’R, 8’’S -dihydroxy-aspernolide E.

#### 2.1.3. Metabolic Profiling of BTL-I in Rats 

**Figure 3:**
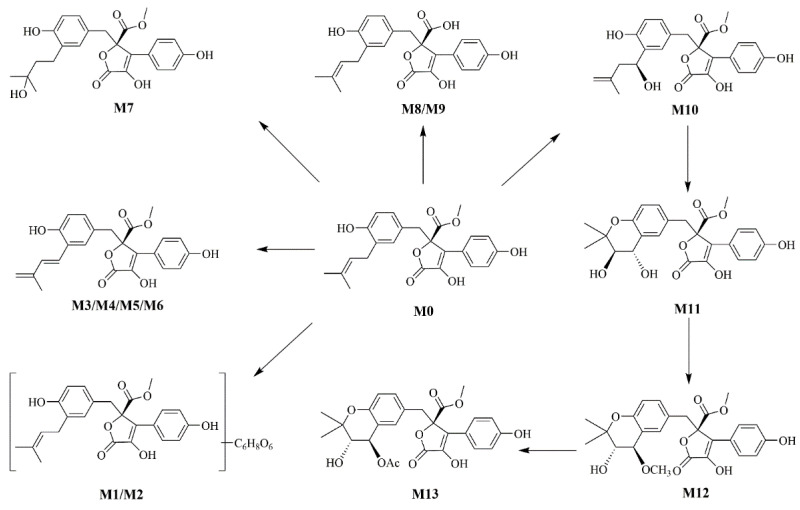
The proposed metabolic pathways of BTL-I in rats.

**Figure 4:**
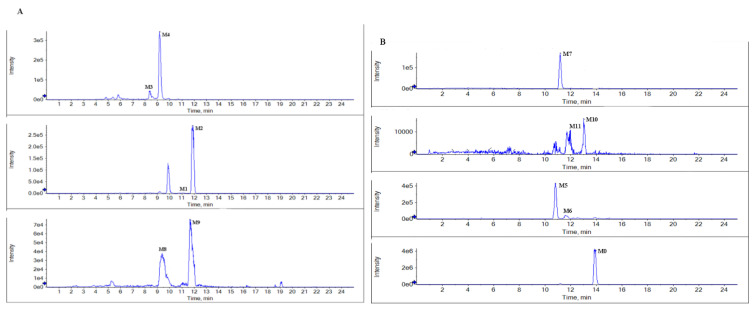
Representative extraction ion chromatograms of the metabolites in rat bile (**A**), and feces (**B**) after the oral administration of BTL-I.

According to the metabolite profiles, the proposed metabolic pathways were predicted, as shown in Figure 3. The elemental compositions, observed and calculated masses, mass errors, and characteristic fragment ions of the proposed metabolites, are summarized in Table 1. The extraction ion chromatographies of the metabolites in rat bile and feces are shown in Figure 4. 

BTL-I was detected in the bile and feces after oral dosing compared with the blank samples. Among the eleven metabolites identified, there was only a trace of the prototype compound detected in plasma and urine after intragastric dosing. Among the metabolites, six metabolites including M1, M2, M3, M4, M8, M9 were detected in the bile samples, and the most abundant metabolites were M2 and M4. Metabolites M5, M6, M7, M10, M11 were detected in the feces, and the most abundant metabolite was M5 and M7. It was thought that BTL-I was poorly absorbed into plasma after oral administration. The absorbed BTL-I was quickly excreted into bile, and metabolized to the metabolites. The result was in agreement with the reported literature [18].

### 2.2. Pharmacokinetic Study 

#### 2.2.1. Method Development

The LC-MS/MS conditions, including parent-product ion transitions and source parameters, were further investigated for analyzing BTL-I and the internal standard (IS), ginsenoside Rg1. Although the response of BTL-I was more sensitive in positive ion mode than that in negative ion mode, the signal was more stable in negative ion mode than that in positive ion mode. In addition, BTL-I could easily form other adduct molecular ions in positive ion mode, for example, adding sodium. Therefore, negative ion mode was adopted for the quantitation of BTL-I in plasma. BTL-I showed the parent ion [M − H]^−^ at *m/z* 423.1, and IS showed the parent ion [M − H] ^−^ at *m/z* 845.6. In product ion scan mode, BTL-I and IS showed product ions at *m/z* 364.1 and 799.5, respectively. Therefore, *m/z* 423.1–364.1 for BTL-I, and *m/z* 845.6–799.5 for IS were applied as MRM transitions. Then, collision energies and other source parameters were optimized to obtain higher sensitivity. 

To obtain the satisfactory shape of the chromatographic peak and retention times, different aqueous phases, including water, 0.1% FA in water, and 5 mM ammonium acetate in water, were compared. The response of BTL-I using 0.1% FA in water was higher than that of the other aqueous phases. Therefore, acetonitrile and 0.1% FA in water were selected as the mobile phase.

#### 2.2.2. Method Validation

**Figure 5:**
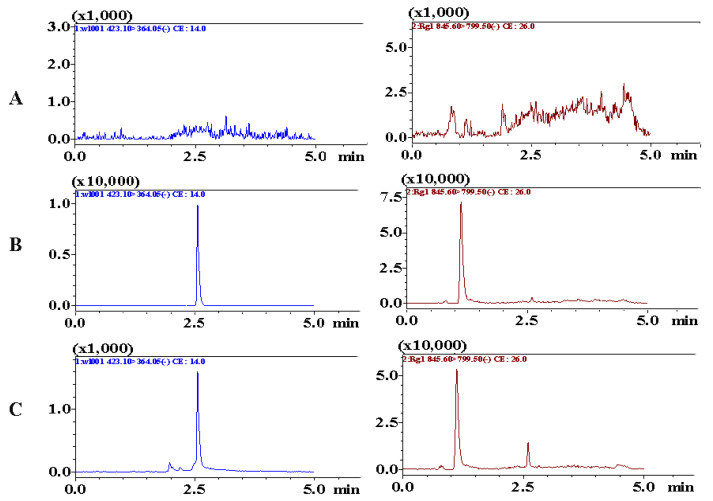
Representative MRM chromatograms of BTL-I (2.51 min) and IS (1.22 min) in (**A**) blank rat plasma, (**B**) blank rat plasma spiked with BTL-I at LLOQ (2 ng/mL) and IS, (**C**) rat plasma samples collected after intragastric administration.

Chromatograms including blank plasma samples, blank samples spiked with analytes, and plasma samples collected after administration, were obtained to assess specificity. As shown in Figure 5, the retention times of BTL-I and IS were 2.51 and 1.22 min, respectively. No interference or co-eluting peaks were observed at the retention times of analytes. The method showed good linearity over the range of 2–500 ng/mL, with a regression coefficient (*r*) of 0.9956. The LLOQ was 2 ng/mL and the LOD was 0.5 ng/mL, which suggested that the method was sensitive enough to determine BTL-I in rat plasma. 

Intra- and inter-precision and accuracy were summarized in Table 2. The results suggested that the concentration of BTL-I in rat plasma could be accurately and precisely measured by the current assay.

The results of the extraction recovery and matrix effect were listed in Table 3. The recovery was over the range of 72.37% to 79.84%. The values of the matrix effect were greater than 85%, indicating that the matrix effect could be ignored.

The stability results of BTL-I under different conditions were listed in Table 4. The data demonstrated that BTL-I was stable during the sample collection, preparation and analysis process. Bias (%) ranging of 4.2 % to 5.1 % in incurred sample stability assay further confirmed the good stability of BTL-I and its metabolites in rat plasma.

#### 2.2.3. Pharmacokinetic Study

**Figure 6:**
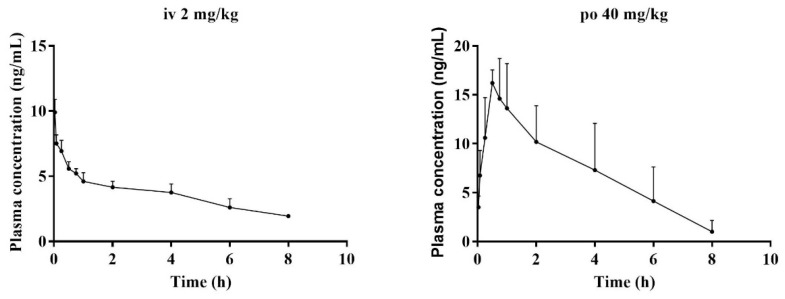
Mean plasma concentration–time curves of BTL-I in rats, after intravenous (2 mg/kg) and intragastric (40 mg/kg) administrations (*n* = 5).

After validation, the method was further applied to study the pharmacokinetic properties of BTL-I in rats after intravenous and oral administration. No obvious abnormalities in the rats’ general conditions were observed. There was no significant increase or decrease in the activity of the rats. The concentrations of BTL-I in rat plasma versus time profiles were depicted in Figure 6, and the pharmacokinetic parameters were listed in Table 5. 

After intravenous administration (2 mg/kg), the concentration of BTL-I at the first time point was extremely low (19.85 ± 1.54 ng/mL). The value of the apparent volume of distribution (Vd) was 1375.46 ± 328.31 L/kg, suggesting that BTL-I was mainly distributed in the organs. BTL-I was quickly eliminated from the plasma, with a T_1/2_ value of 1.36 ± 0.25 h. After oral administration, BTL-I was rapidly absorbed into plasma and detected at 5 min after oral administration. BTL-I reached the peak at 0.69 ± 0.24 h, with a C_max_ value of 17.97 ± 1.36 μg/L. BTL-I was quickly eliminated from the plasma, with a T_1/2_ value of 1.23 ± 0.22 h. The AUC_0-∞_ after oral administration and intravenous administration were 60.5 ± 26.85 ng*h/mL and 48.09 ± 6.68 ng*h/mL, respectively. The oral absolute bioavailability was calculated as 6.29%. According to the current result, the metabolites with a high abundance in tissue deserve further study.

## 3. Materials and Methods

### 3.1. Chemicals and Reagents

BTL-I was isolated from a deep-sea-derived fungus and identified by Dr. Chunlan Xie in the Third Institute of Oceanography, Ministry of Natural Resources, as previously reported [14]. The chemical structure was confirmed by nuclear magnetic resonance (NMR) spectroscopy (Figures S1 and S2). The purity was above 98%, as determined by HPLC. Ginsenoside Rg1 was purchased from Shanghai R&D Centre for Standardization of Chinese Medicines (Shanghai, China). HPLC grade of acetonitrile and MeOH were purchased from Fisher Scientific Co. (Santa Clara, CA, USA). Ultra-high purified water was prepared using a Milli-Q water-purification system (Millipore Co., Billerica, MA, USA). The other chemicals were purchased from Sinopharm chemical reagent Co., Ltd. (Shanghai, China).

### 3.2. Animals, Drug Administration and Sampling

Male Sprague-Dawley rats (230–250 g) were provided by SLEK Laboratory Animal Co., Ltd. (Shanghai, China). The animals were housed in an environmentally controlled breeding room for 7 days, and fed with standard laboratory food and water ad libitum, except for a fasting period 12 h prior to experiment. The animal experiments were approved by the Animal Ethics Committee of Shanghai University of Traditional Chinese Medicine (No: PZSHUTCM20107008). 

For pharmacokinetic study, 12 rats were randomly assigned into two groups (*n* = 6). BTL-I (2 mg/mL) was dissolved in 5% dimethylsulfoxide, 5% tween-80 and 90% saline. Animals were administrated with BTL-I intravenously (2 mg/kg) and orally (40 mg/kg). Venous blood samples were collected into heparinized tubes from the tail at pre-dose and pre-defined times (2 (intravenous only), 5, 15, 30 min and 1, 2, 4, 6, 8, 24 h) after administration. The plasma sample was harvested by centrifuging blood at 4000× *g* for 10 min, and then stored at −20 °C until further preparation. 

For the metabolism study, 12 rats were randomly divided into four groups (*n* = 3): plasma, urine, bile and feces groups. Animals were administrated with BTL-I orally (50 mg/kg). Blank blood samples were collected before administration and the collected blood samples, at different time points (0.25, 0.5, 1, 2, 4, 8 h), were pooled in equal volumes. The pooled blood samples were centrifuged at 4000× *g* for 10 min to obtain the plasma samples. Urine, bile and feces samples were collected 4 h pre-dose and 0–24 h post-dose. All the samples were stored at −20 °C prior to analysis.

### 3.3. Sample Preparation

#### 3.3.1. Sample Pretreatment

For pharmacokinetic study, 150 μL methanol-containing IS (100 ng/mL) was added to a portion of 50 μL plasma samples. The mixture was vortexed for 30 s and centrifuged at 13,000 rpm for 10 min to precipitate the proteins. A 150 μL aliquot of the supernatant was mixed with an equal volume of water. After vortexed for 30 s, 5 μL aliquot was analyzed by LC-MS/MS. 

For the metabolism study, samples were prepared by protein precipitation to enrich the concentrations of metabolites. 4 mL MeOH was added to 1 mL plasma/1 mL urine/1 mL bile sample in tubes, and then vortexed for 2 min. After that, the mixture was centrifuged at 13,000 rpm for 10 min. The supernatant was transferred to a new tube and evaporated to dryness under a stream of nitrogen at room temperature. The residue was reconstituted by 200 µL 50% methanol. Afterwards, it was vortexed for 30 s and centrifuged at 13,000 rpm for 10 min; then, 5 µL of the supernatant was analyzed by UHPLC–Q-TOF-MS system. Feces sample (100 mg) was homogenized in 4 folds volumes of saline, the homogenate was extracted with 900 µL of methanol, vortexed for 1 min, and centrifuged at 13,000 rpm for 10 min. The supernatant was transferred to a clean tube and evaporated to dryness under a stream of nitrogen. The residue was reconstituted by 200 µL 50% methanol, vortexed for 30 s and centrifuged at 13,000 rpm for 10 min. Then 5 µL of the supernatant was analyzed by the UHPLC–Q-TOF-MS system.

#### 3.3.2. Preparation of Standard Curve and Quality Control (QC) Samples

BTL-I standard was weighed accurately and dissolved in DMSO to prepare stock solution (1 mg/mL). Different concentrations of working solutions (2, 10, 20, 100, 250, 500 ng/mL) were prepared by serial diluting the stock solution with MeOH. The QC samples were prepared similarly to the calibration standard samples with concentration levels of 5, 50, and 400 ng/mL for the low, medium, and high levels, respectively. A working solution of IS at the concentration of 100 ng/mL was prepared in the same manner.

### 3.4. Instrument and LC–MS Conditions

#### 3.4.1. LC–MS/MS Condition

For the pharmacokinetic study, the LC–MS/MS instruments were composed of a Shimadzu LC system equipped with SIL-30AC autosampler LC-30AC binary pump, a CTO-20AC column oven, and a 8050 triple quadrupole mass spectrometer, coupled with an electrospray ionization source. The chromatographic separation was conducted on an ACQUITY UPLC HSS T3 column (100 × 2.1 mm, 1.8 μm) with the column temperature was at 40 °C. The mobile phases were composed of 0.1% formic acid aqueous solution (A), and acetonitrile (B). The gradient elution was set as follows: 0–0.5 min, 5% B; 0.5–1 min, 5–80% B; 1–3 min, 80% B; 3–5 min, 5% B. The flow rate was set at 0.4 mL/min and the injection volume was set at 5 μL.

Source parameters were optimized as follows: interface temperature, 300 °C; capillary voltage, 500 V; gas flow, 10 L/min; nebulizer gas, 3 L/min. The multiple reaction monitoring (MRM) mode was selected for quantitative analysis in negative ion mode. The precursor-to-product ion transitions were *m/z* 423.1–364.1 for BTL-I and *m/z* 845.6–799.5 for IS.

#### 3.4.2. UHPLC–Q-TOF-MS Condition

For the metabolic profiling study, a Shimadzu 3000 UHPLC system combined with an AB SCIEX 5600 QTOF mass spectrometer (AB Sciex) was applied for the analysis of the metabolites. The chromatographic separation was performed on an ACQUITY UPLC HSS T3 column (100 × 2.1 mm; 1.8 μm). The mobile phase consisted of water containing 0.1% formic acid (A), and acetonitrile (B), at a flow rate of 0.4 mL/min. The gradient elution was set as follows: 5–20% B at 0–4 min; 20–40% B at 4–10 min; 40–60% B at 10–15 min; 60–80% B at 15–16 min; 80–95% B at 16–18 min; 95% B at 18–20 min, and finally 5% B at 20–25 min. The injection volume was 2 µL. The ESI source parameters were set as follows: spray voltage, 3.0 kV; capillary temperature, 300 °C; sheath gas, 40 arb; auxiliary gas, 10 arb; S-Lens voltage, 50 V. The data was acquired over the range of *m/z* 100–1000 Da.

### 3.5. Method Validation 

The method validation was operated according to the Bioanalytical method validation guidance for industry, issued by the United States Food and Drug Administration (FDA). The items of validation included selectivity, carry-over, linearity, precision and accuracy, matrix effect, extraction recovery, and stability.

#### 3.5.1. Selectivity and Carry-Over Effect

The selectivity of the method was evaluated by analyzing blank plasma samples from six different individuals, blank plasma spiked with BTL-I and IS, as well as the plasma samples obtained after oral administration. 

The carry-over effect was assessed by analyzing two processed, blank plasma samples after the highest concentration point of the calibration curve. The response in the blank sample should be less than 20% of LLOQ for BTL-I and 5% for IS. 

#### 3.5.2. Linearity and Lowest Limit of Quantitation (LLOQ)

A standard curve was plotted using the ratio of BTL-I peak area/IS peak area against sample concentrations, including six different concentrations. The linearity of the method was assessed by the standard curve. The LLOQ was defined as the concentration at which the ratio of signal to noise was not less than 10.

#### 3.5.3. Precision and Accuracy

The precision was expressed as relative standard deviation (RSD%) and the accuracy was relative error (RE%). The intra- and inter-day precision and accuracy were assessed by calculating the LLOQ and QC samples (2, 5, 50, 400 ng/mL) in six replicates on the same day and on three consecutive days, respectively. As required, variation should not exceed 15% for both precision and accuracy. 

#### 3.5.4. Matrix Effect and Extraction Recovery 

The matrix effect and extraction recovery were evaluated by the peak area ratio of BTL-I to IS at three QC levels. The specific evaluating processes were as follows: matrix effect was evaluated by peak area ratio (A) of BTL-I to IS, dissolved in the supernatant of the processed blank plasma, compared with the corresponding peak area ratio (B) of BTL-I to IS, in the pure water at three QC levels. The ratio of A to C was used to evaluate the extraction recovery, where C represented the peak area ratio of BTL-I to IS dissolved in the spiked blank plasma. 

#### 3.5.5. Stability

The stability of the QC samples was investigated by analyzing the samples under four storage conditions, including being kept at room temperature for 8 h, being stored at −20 °C for 15 days, being kept at 4 °C for 24 h, and being subjected to three freeze–thaw cycles. As required, RSD should not exceed 15%. 

### 3.6. Data Analysis

The UHPLC–MS/MS data was acquired and processed using Labsolutions^TM^ software (Shimadzu, Japan). The pharmacokinetic parameters, including the area under the concentration–time curve (AUC), clearance (Cl), volume of distribution (Vd), mean residence time (MRT), peak concentration (C_max_), the time at the peak concentration (T_max_) and the elimination of half-lives (T_1/2_), were calculated using the PK Solutions 2.0^TM^ (Summit Research Services, USA) by a non-compartmental model. The absolute bioavailability was calculated based on the equation: F (%) = (AUCp.o. × Dosei.v.)/(AUCi.v. × Dosep.o.) × 100%.

Raw UHPLC–Q-TOF-MS data files were acquired by Analyst software (Version 2.3.1, AB sciex, CA, USA) and imported into Peakview (Version 1.6, AB sciex, CA, USA) software to identify the metabolites of BTL-I. For comprehensive profiling, the whole process was divided into automatic prediction and manual validation. In the step of automatic prediction, the high resolution mass spectrometry data was obtained by inputting the chemical formula and its metabolic intermediates with the ionization set as [M + H]^+^ or [M + Na]^+^ and [M − H]^−^ or [M + HCOO]^−^. Then, candidate metabolites were predicted by the software based on the typical metabolic reactions of templates. The step of manual validation was achieved by extracting ion chromatograms to screen candidate metabolites with the mass tolerance set to 5 ppm. Moreover, MS^2^ fragment ions were extracted for the structural confirmation of candidate metabolites.

## 4. Conclusions

In the present study, *in vivo* pharmacokinetics and the metabolism of BTL-I were investigated. A total of eleven metabolites were detected in rats by UHPLC–Q-TOF-MS. The major metabolic pathways were oxidative and glucuronide conjugation. In the pharmacokinetic study, BTL-I was quickly eliminated from the plasma. The oral bioavailability was approximately 6.29%. The information gained in this study was helpful for us to understand the pharmacological actions of the drug.

## Figures and Tables

**Table 1:** Mass spectral data of BTL-I (M0) and its metabolites (M1-M11). U, urine; F, feces; B, bile.

	*t_R_*	*Measured m/z*	Calculated *m/z*	Formula	Product ions	Error (ppm)	Source
M0	13.87	425.1598	425.1600	C_24_H_25_O_7_	393.1306, 331.1369, 275.0701, 175.1124	−0.5	B F
M1	11.08	601.1906	601.1910	C_30_H_33_O_13_	425.1596, 393.1300, 331.1301, 175.1111	−0.7	B U
M2	11.87	601.1906	601.1910	C_30_H_33_O_13_	425.1606, 393.1301, 331.1334, 175.1115	−0.7	B
M3	8.42	423.1440	423.1444	C_24_H_23_O_7_	391.1141, 345.1099, 329.1217, 317.1183, 173.0970	−1.0	B
M4	9.19	423.1440	423.1444	C_24_H_23_O_7_	391.1141, 345.1121, 329.1180, 317.1204, 173.0976	−1.0	B
M5	10.82	423.1440	423.1444	C_24_H_23_O_7_	345.1134, 329.1214, 275.0971, 173.0961	−1.0	F
M6	11.63	423.1440	423.1444	C_24_H_23_O_7_	345.1134, 329.1214, 275.0971, 173.0961	−1.0	F
M7	11.17	443.1698	443.1706	C_24_H_27_O_8_	349.0429	−1.8	F
M8	9.38	411.1443	411.1444	C_23_H_23_O_7_	275.0743, 175.1098	−0.2	B
M9	11.71	411.1443	411.1444	C_23_H_23_O_7_	393.1261, 331.1387, 275.0768, 175.1133	−0.2	B
M10	13.03	441.1525	441.1549	C_24_H_25_O_8_	363.1112	−5.4	F
M11	11.96	457.1506	457.1498	C_24_H_25_O_9_	363.1112	1.8	F

**Table 2:** Precision and accuracy of BTL-I in rat plasma (*n* = 6).

Concentration (ng/mL)	Intra-Day		Inter-Day	
	Precision (R.S.D., %)	Accuracy	Precision (R.S.D., %)	Accuracy
2	2.95	87.95 ± 5.62	7.25	86.45 ± 7.33
5	3.87	101.23 ± 4.06	6.41	91.52 ± 6.31
200	1.23	91.44 ± 2.95	5.12	90.89 ± 4.88
400	3.18	96.83 ± 3.33	4.14	92.77 ± 5.36

**Table 3:** Extraction recovery and matrix effect of BTL-I in rat plasma (*n* = 6).

	Nominal Conc. (ng/mL)	Matrix Effect (%)	Extraction Recovery (%)
BTL	5	86.13 ± 6.33	75.09 ± 4.29
	200	88.29 ± 5.14	72.37 ± 7.22
	400	92.14 ± 3.19	79.84 ± 6.15
IS	20	85.12 ± 4.47	80.17± 5.09

**Table 4:** Stability of BTL-I under different storage conditions (*n* = 6).

**Stability**	**Nominal** **Concentrations (ng/mL)**	**Measured** **Concentrations (ng/mL)**	**Accuracy R.E. (%)**	**R.S.D. (%)**
Short term	5	4.82 ± 0.33	96.36 ± 6.67	6.85
	200	189.71 ± 11.15	94.86 ± 5.58	5.87
	400	405.27 ± 18.51	101.32 ± 4.63	4.57
Long term	5	4.78 ± 0.21	95.86 ± 4.27	4.39
	200	190.71 ± 13.14	95.36 ± 6.57	6.89
	400	411.27 ± 12.44	102.82 ± 3.11	3.02
Post-preparative	5	5.19 ± 0.42	103.80 ± 8.09	7.50
	200	197.51 ± 12.49	98.76 ± 7.38	7.45
	400	401.39 ± 23.84	100.35 ± 5.88	5.91
Freezing and thawing cycles	5	5.07 ± 0.32	101.50 ± 6.40	6.50
	200	195.51 ± 21.49	97.76 ± 10.75	10.45
	400	403.29 ± 18.89	100.83 ± 4.72	5.11

**Table 5:** Pharmacokinetic parameters of BTL-I in rat plasma after oral (40 mg/kg) and intravenous (2 mg/kg) administration (*n* = 5).

	Unit	Intravenous	Oral
AUC_0-t_	ng*h/mL	45.13 ± 3.96	57.93 ± 26.11
AUC_0-∞_	ng*h/mL	48.09 ± 6.68	60.5 ± 26.85
T_max_	h		0.69 ± 0.24
C_max_	ng/mL		17.97 ± 1.36
Cl	L/h/kg	272.11 ± 66.64	292.64 ± 171.51
Vd	L/kg	1375.46 ± 328.31	5730.25 ± 2415.75
MRT	h	5.68 ± 1.48	2.88 ± 0.55
T_1/2_	h	1.36 ± 0.25	1.23 ± 0.22
F	%		6.29

## Data Availability

Data available on request due to restrictions e.g., privacy or ethical.

## Supplementary Materials

The following are available online at https://www.mdpi.com/article/10.3390/md20010011/s1, Figures S1–S4: ^1^H, ^13^C, HSQC, and HMBC NMR spectra of BTL-I.
