# Supplementary material for: Pharmacokinetics and Metabolism Study of Deep-Sea-Derived Butyrolactone I in Rats by UHPLC–MS/MS and UHPLC–Q-TOF-MS
Source: Mar Drugs. 2021 Dec 22;20(1):11. doi: 10.3390/md20010011 (PMC8780701; doi:10.3390/md20010011)
Supplement: Supplementary file 1 [file marinedrugs-20-00011-s001.zip › marinedrugs-1481670-supplementary.pdf]

Supplementary Materials

Pharmacokinetics and Metabolism Study of Deep-Sea-Derived Butyrolactone I in Rats by UHPLC-MS/MS and UHPLC-Q-TOF-MS

Liang Wu [1], Chun-Lan Xie [2], Xian-Wen Yang [2,*] and Gang Chen [1,*]

[1] School of Pharmacy, Fudan University, Shanghai, China; yhdhospital@163.com

[2] Key Laboratory of Marine Biogenetic Resources, Third Institute of Oceanography, Ministry of Natural Resources, 184 Daxue Road, Xiamen, 361005 Fujian, China; xiechunlanxx@163.com

* Correspondence: yangxianwen@tio.org.cn (X.-W.Y.); hhcyy_edu12@163.com (G.C.); Tel.: +86-592-219-5319 (X.-W.Y.); +86-21-51980168 (G.C.).

Content

Figure S1. ^{1}H NMR spectrum of **1** in MeOD.

Figure S2. ^{13}C NMR spectrum of **1** in MeOD.

Figure S3. HSQC NMR spectrum of **1** in MeOD.

Figure S4. HMBC NMR spectrum of **1** in MeOD.

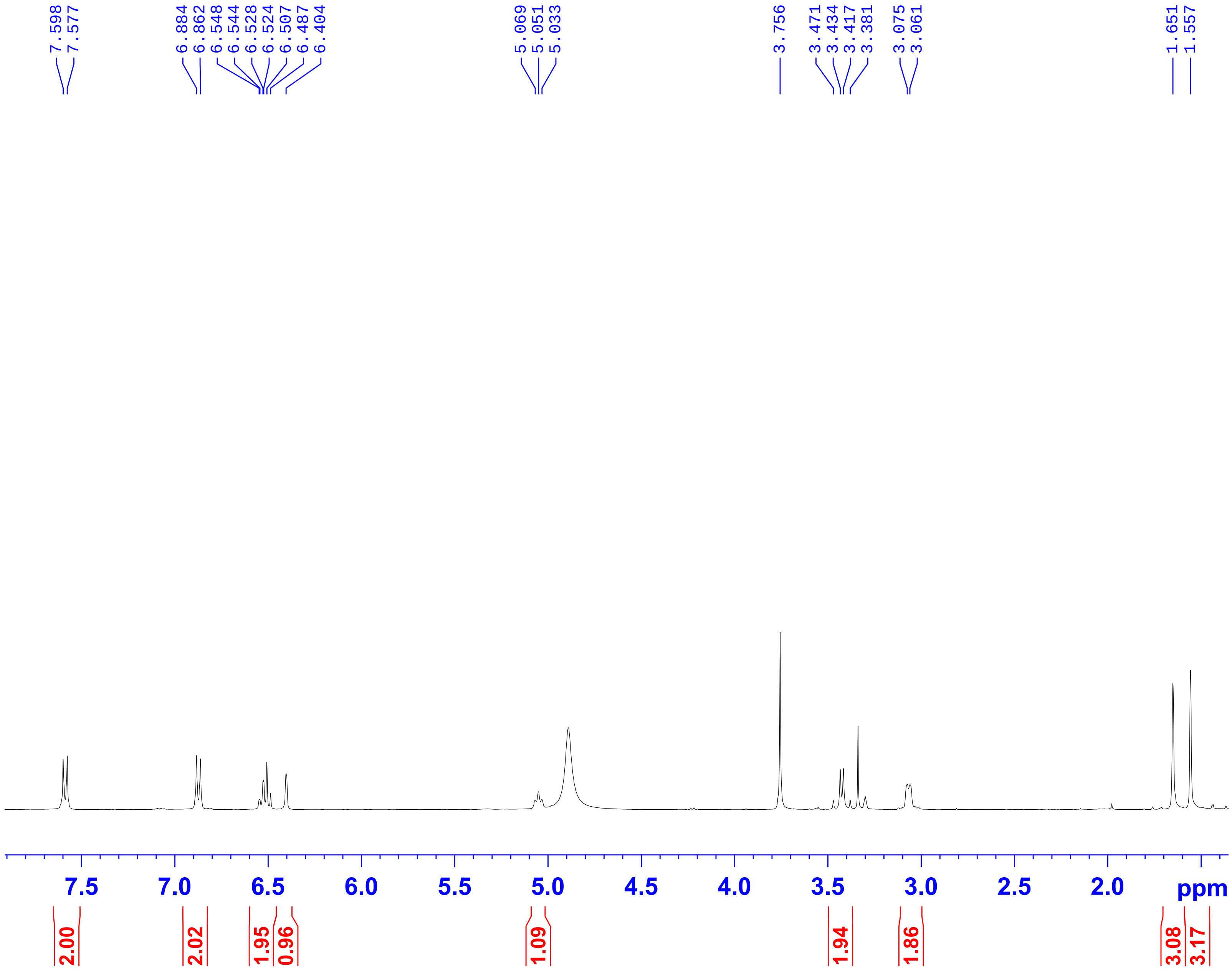

Figure S1. ^{1}H NMR spectrum of **1** in MeOD.

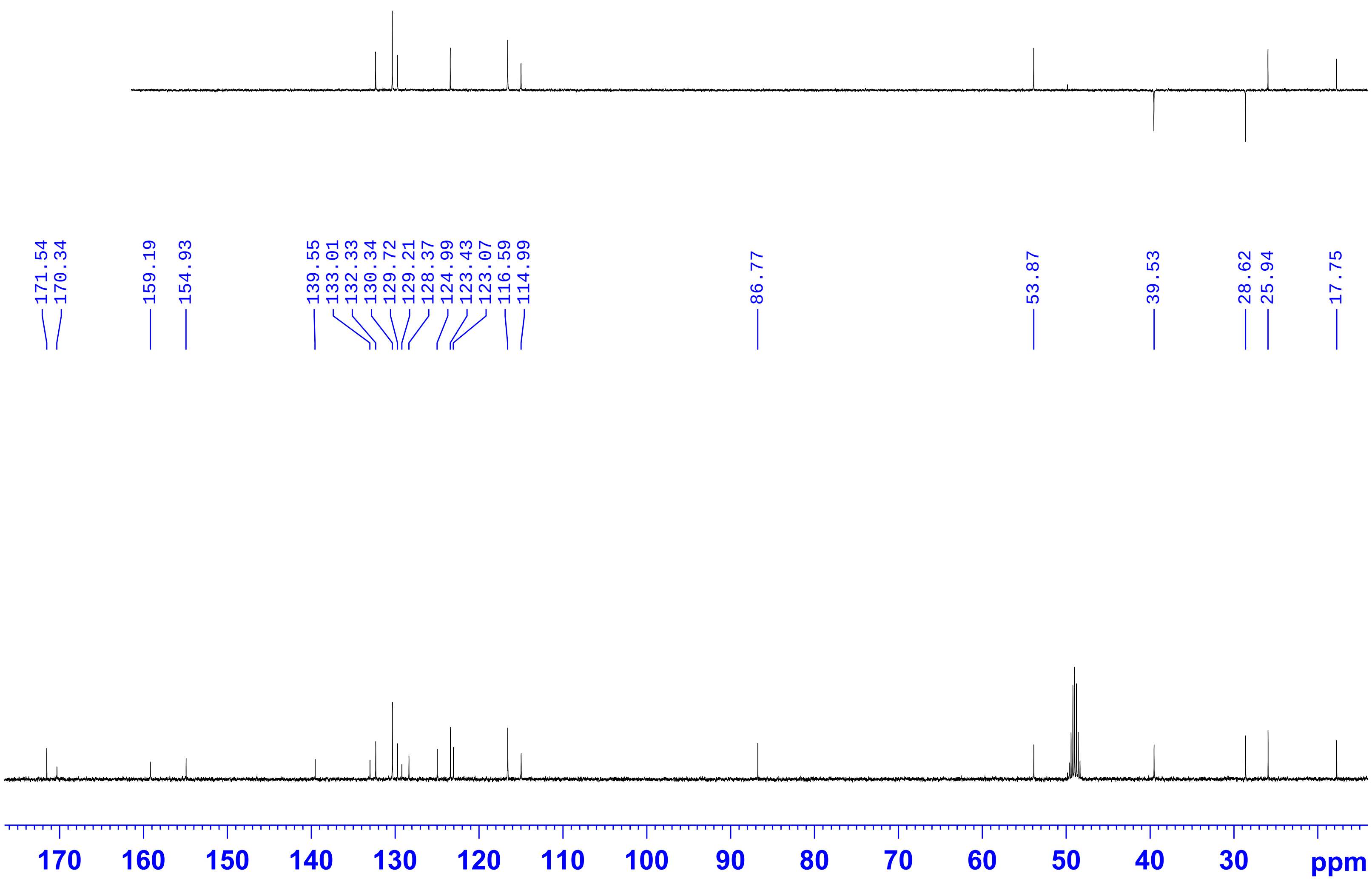

Figure S2. ^{13}C NMR spectrum of **1** in MeOD.

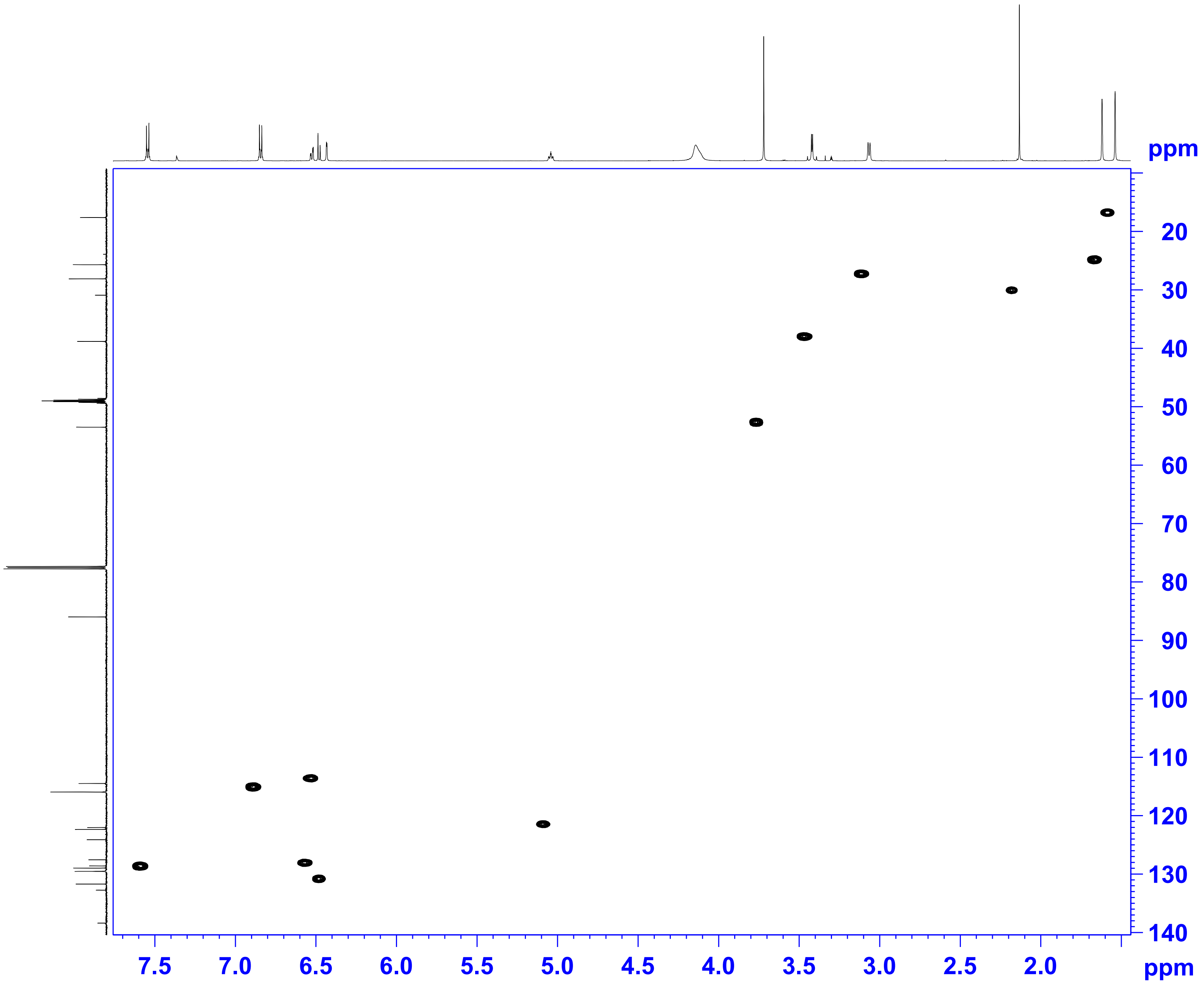

Figure S3. HSQC NMR spectrum of **1** in MeOD.

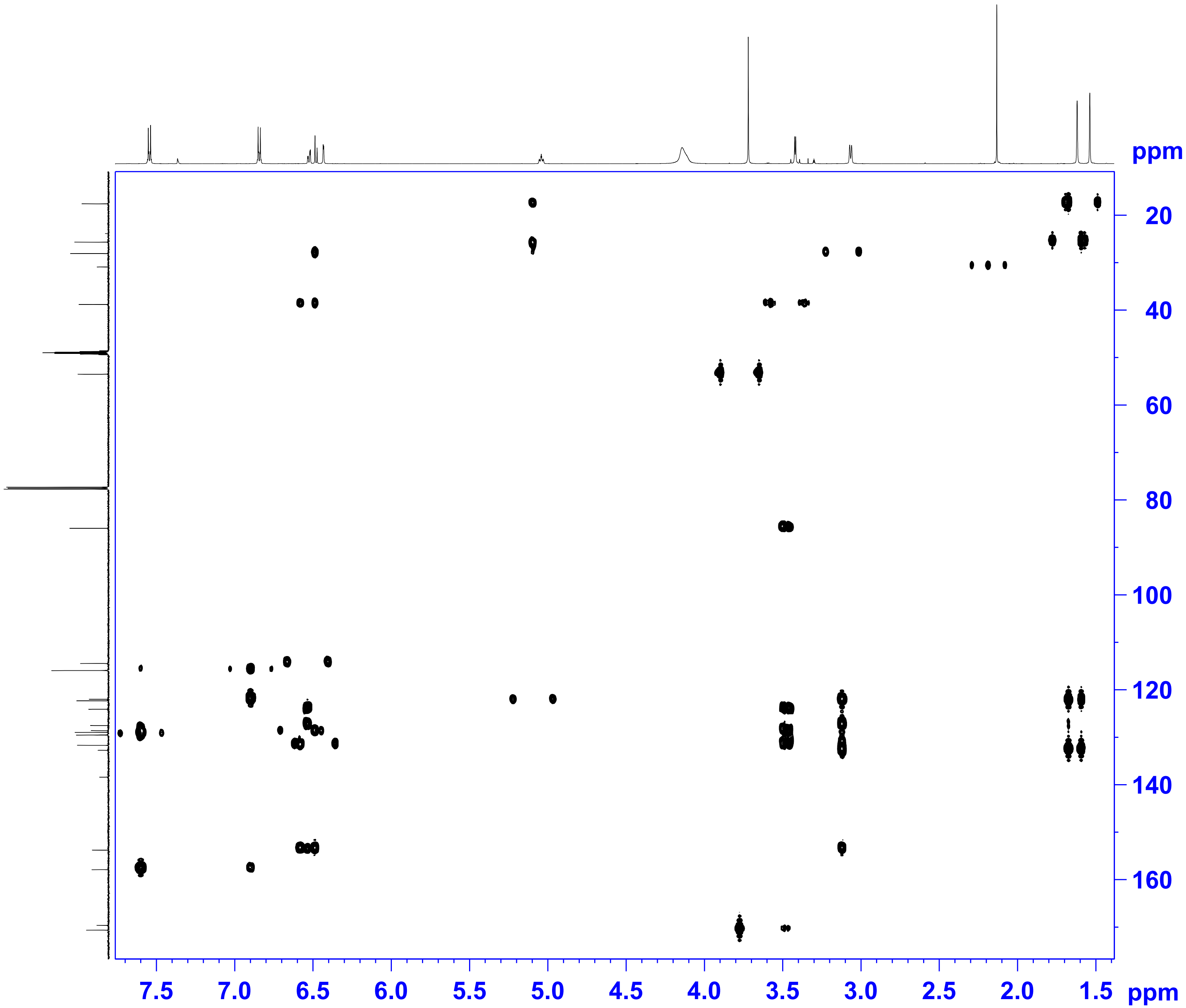

Figure S4. HMBC NMR spectrum of **1** in MeOD.
